# Study protocol of OmegaROP-2 prospective study: expression of placental fatty acid receptors in preterm newborns with retinopathy of prematurity

**DOI:** 10.1186/s12886-023-03156-0

**Published:** 2023-10-06

**Authors:** Chloé Carré, Niyazi Acar, Alejandra Daruich, Stéphane Grégoire, Lucy Martine, Bénédicte Buteau, Serge Aho, Petra Eid, Louis Arnould, Alain Marie Bron, Marine Driessen, Elsa Kermorvant, Emmanuel Simon, Catherine Creuzot-Garcher, Pierre-Henry Gabrielle

**Affiliations:** 1 Department of Ophthalmology, Dijon University Hospital, 14 Rue Paul Gaffarel, 21000 Dijon, France; 2 Eye and Nutrition Research Group, Centre Des Sciences du Goût Et de L’Alimentation, AgroSup Dijon, CNRS, INRAE, Université Bourgogne Franche-Comté, Dijon, France; 3 Department of Ophthalmology, Necker Hospital, Paris, France; 4 Department of Epidemiology & Biostatistics, Dijon University Hospital, Dijon, France; 5 Department of Gynecology, Necker Hospital, Paris, France; 6 Department of Neonatology, Necker Hospital, Paris, France; 7 Department of Gynecology, Dijon University Hospital, Dijon, France

**Keywords:** Docosahexaenoic acid, FATP, Placental fatty acid receptor, Retinopathy of prematurity

## Abstract

**Background:**

Incomplete vascularization of the retina in preterm infants carries a risk of retinopathy of prematurity (ROP). Progress in neonatal resuscitation in developing countries has led to the survival of an increasing number of premature infants, resulting in an increased rate of ROP and consequently in visual disability. Strategies to reduce ROP involve optimizing oxygen saturation, nutrition, and normalizing factors such as insulin-like growth factor 1 and n-3 long-chain polyunsaturated fatty acids (LC-PUFA). Our previous study, OmegaROP, showed that there is an accumulation or retention of docosahexaenoic acid (DHA) in mothers of infants developing ROP, suggesting abnormalities in the LC-PUFA placental transfer via fatty acid transporting proteins. The present study aims to better understand the LC-PUFA transport dysfunction in the fetoplacental unit during pregnancy and to find a novel target for the prevention of ROP development.

**Methods:**

The study protocol is designed to evaluate the correlation between the expression level of placental fatty acid receptors and ROP occurrence. This ongoing study will include 100 mother-infant dyads: mother-infant dyads born before 29 weeks of gestational age (GA) and mother-infant dyads with full-term pregnancies. Recruitment is planned over a period of 46 months. Maternal and cord blood samples as well as placental tissue samples will be taken following delivery. ROP screening will be performed using wide-field camera imaging according to the International Classification of ROP consensus statement.

**Discussion:**

The results of this study will have a tangible impact on public health. Indeed, if we show a correlation between the expression level of placental omega-3 receptors and the occurrence of ROP, it would be an essential step in discovering novel pathophysiological mechanisms involved in this retinopathy.

**Trial registration:**

NCT04819893.

## Background

Physiologically, the retina does not have blood vessels until the fourth month of gestation, and the temporal periphery is normally fully vascularized by 1 month after birth. Incomplete vascularization of the retina in preterm infants carries a risk of retinopathy of prematurity (ROP) [1]. Therefore, screening for ROP is recommended in infants born before 30 weeks of gestational age (GA) or those weighing less than 1500 g [2]. ROP is a pathological process in the immature retina leading to retinal neovascularization complications such as tractional retinal detachment, which results in subsequent visual loss. The incidence of ROP in developed countries is highly variable and ranges from 6 to 34% [3]. Despite improvements in controlling risk factors, ROP remains a leading cause of blindness. Although progress in neonatal resuscitation has led to the survival of an increasing number of premature infants, especially in developing countries, the rates of ROP and resulting visual loss have risen simultaneously [3–5]. High oxygenation targets are associated with decreased mortality, but hyperoxia inhibits the development of retinal vascularization. Subsequently, the increased retinal metabolic activity triggers growth factor-induced retinal vasoproliferation in the poorly vascularized retina [6]. Depending on the location and stage of the ROP, intravitreal vascular endothelial growth factor (VEGF) inhibitor or laser ablation of non-vascularized retinal areas reduces the risk of blindness related to ROP have shown satisfactory outcomes, still some treated infants do not achieve good long-term visual acuity due to several other complications related to the disease. Therefore, prevention through the control of risk factors is more effective than late treatment of neovascularization. Strategies to reduce ROP involve optimizing oxygen saturation, nutrition, and normalizing factors such as insulin-like growth factor 1 (IGF-1) and omega 3 (n-3) long-chain polyunsaturated fatty acids fatty acids (LC-PUFA) [7].

The mechanisms of normal and pathological retinal vascular development have already been extensively studied in ROP. Among the factors influencing the abnormal vascularization process in ROP are PUFAs and/or their derivatives, which appear to be potent retinal vascular growth regulators. For example, n-3 PUFAs have been shown to prevent pathological angiogenesis in age-related macular degeneration [8–12], retinal vascular damage in diabetes [13], and abnormal retinal vascular development in an animal model of ROP (model of oxygen-induced retinopathy) [14].

Docosahexaenoic acid (DHA) is protective in experimental models, but its administration as part of parenteral nutrition has yielded inconsistent results. According to the literature, few studies have been performed in vivo with extremely preterm infants. Some studies have not found a correlation between ROP development and DHA supplementation [15, 16], whereas others have [17–19]. According to Berbade-Garcia and colleagues, it seems that LC-PUFA supplementation is correlated with a lower occurrence of severe ROP [20].

Concerning molecular signaling pathways, PUFAs modulate IGF-1 activation pathways [21] and VEGF-induced endothelial cell proliferation [22, 23]. These signaling pathways are implicated in the pathophysiology of ROP [24–26]. Postmortem studies have shown that the fatty acid composition of erythrocytes is correlated with that of nerve structures in children [27]. The pre- and postnatal developmental period is the most active phase of LC-PUFA incorporation into the central nervous system, including the retina [28].

Since retinal lipid analysis in humans is inconceivable due to its invasive nature, biochemists use circulating biomarkers of retinal LC-PUFAs. The lipid composition of erythrocyte membranes is considered a more reliable reflection than plasma since lipids in red blood cells are less sensitive to external factors. The omega-3 LC-PUFA composition of erythrocyte membranes does not appear to vary significantly in healthy children during the last weeks of gestation (4.7 ± 1.3% of total fatty acids for DHA at 24 GA [29], 5.2 ± 0.7% at 29 GA [30], 4.6 ± 0.4% at 33 GA [31], and 4.1% with a CI = [1.71–4.86] at 29 GA [32]). DHA is essential for fetal development and cannot be synthesized by the fetus. During pregnancy, fatty acids (FAs) are transferred from the maternal blood to the fetal blood via the placenta and the umbilical cord. At the microvillous membrane of the placenta, lipoprotein lipase induces the catabolism of triglycerides from the maternal blood, followed by placental uptake of free FA products. LC-PUFAs cross the microvillous membrane, the syncytium, and the basement membrane to reach the fetal blood circulation. The transport of nutrients and solutes across the syncytiotrophoblast occurs through several passive and active processes, including flow-limited diffusion, transcellular diffusion, protein-mediated transfer, and endocytosis/exocytosis. Selective transport of long-chain fatty acids occurs via specific transporters called fatty acid-binding protein (FABP) and fatty acid-transporting protein (FATP). These proteins have a major role in the transport of Fas [33, 34]. FATP1 and FATP4 are frequently studied in placental tissue since their expression is correlated with DHA levels in maternal plasma, cord blood, and placental phospholipids, suggesting an essential role in the transfer of LC-PUFA [35]. Recent research is beginning to uncover the mechanisms of DHA transmembrane and intracellular transport in the placenta. It is suggested that maternal health and nutrition during pregnancy could be important in determining FA transport and binding protein expression and, thereby, essential FA delivery to the fetus. Further knowledge in this domain may be the first step in developing targeted interventions to help optimize fetal retinal growth and limit the incidence of ROP.

Our previous study, Omega-ROP, took place at Dijon University Hospital’s ophthalmology and neonatology departments and the National Research Institute for Agriculture, Food and the Environment (INRAE) between July 2015 and January 2018. This project confirmed previously published reports that the bioavailability of circulating (erythrocyte) LC-PUFAs was different in premature newborns developing ROP than in those without ROP [32]. Interestingly, it also showed that blood omega-3 LC-PUFA levels in mothers of premature newborns varied in opposite directions to their newborns. Mothers of newborns developing ROP had increased erythrocyte DHA levels above the normal range found in middle-aged women [36], while the levels of their respective newborns were abnormally low. Thus, our data suggest a potential accumulation or retention of DHA in mothers of infants developing ROP due to a dysfunction in LC-PUFA trans-placental transfer [32]. We decided to continue and extend our research collaboration based on these findings. Therefore, an amendment to collaborate with Necker Hospital in Paris was made for the study to be a multicenter trial.

The present study aims to better understand the underlying cellular and molecular mechanisms by assessing the association between placental FA receptor expression levels and the incidence of ROP in newborns. In addition, we will study the association between the LC-PUFA content of cord blood and maternal blood with the expression level of placental PUFA receptors and their correlation with ROP severity.

## Methods

### Study design

Initially, the OMEGAROP-2 clinical trial (NCT04819893) was a single-center prospective cohort study. However, ROP is an uncommon pathology, with an incidence of approximately 20% in preterm newborns under 29 GA. Therefore, our protocol was amended to extend recruitment from 22 to 46 months at the Dijon University Hospital Maternity Department and to make the study a multicenter project with the collaboration of the ophthalmology and gynecology departments of Necker Hospital, Paris, France, to increase the number of participants.

### Study population and schedule

**Fig. 1:**
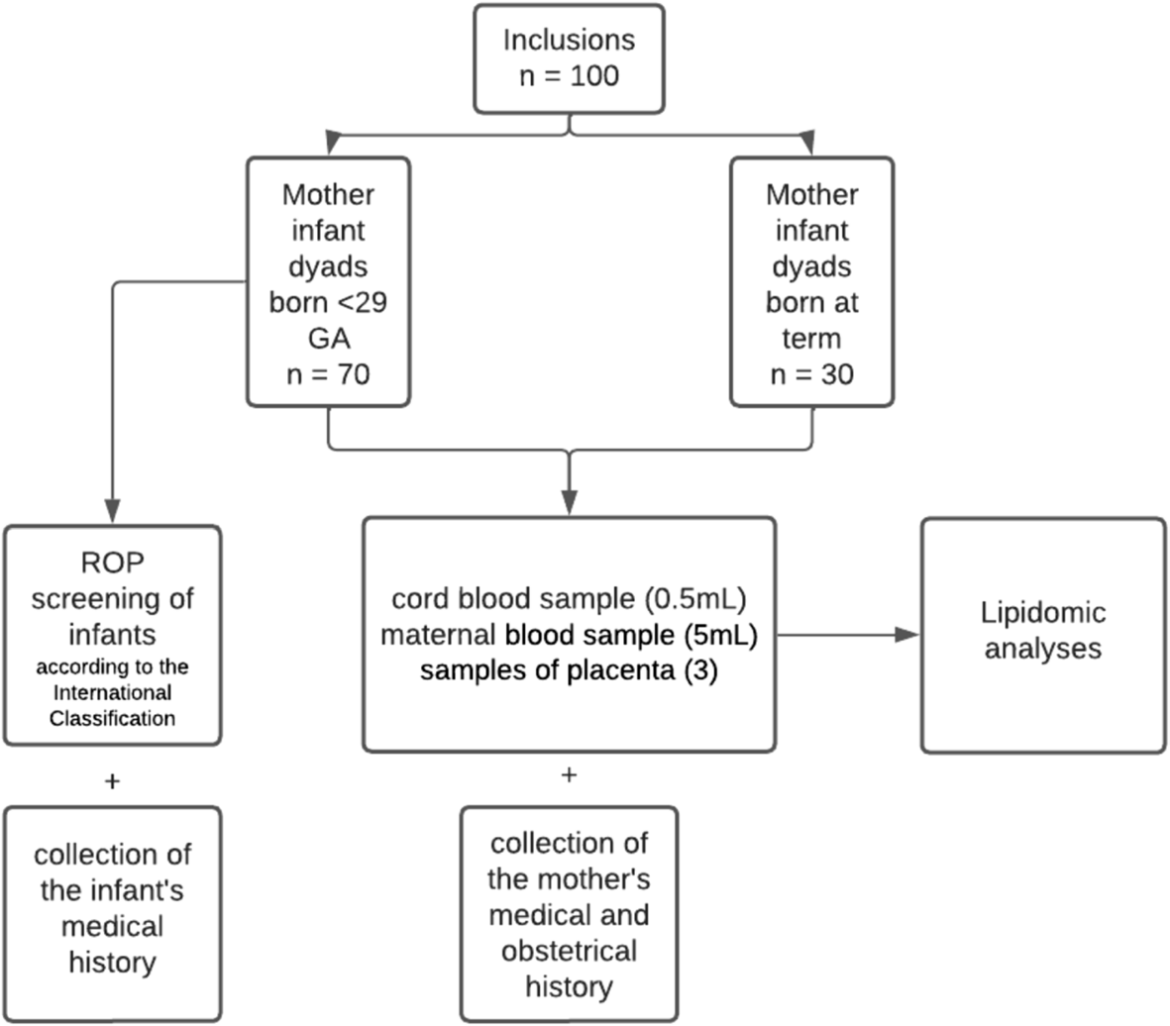
Flowchart of the OmegaROP-2 study

The design of the study is illustrated in Fig. 1.

For mother-infant dyads born before 29 GA, screening will be performed in the Pathological Pregnancy Department. Screening will be carried out in the delivery room for patients delivering at full-term. First, the investigating physician provides the patient with information and answers questions about the research study’s purpose and requirements. They also specify the patient’s rights when participating in biomedical research and verify the eligibility criteria. Second, the investigator obtains the patient’s freely given, informed, and written consent. Finally, the investigator provides a copy of the informed consent form according to article L1122-1 of the Public Health Code. Patients who do not consent to the trial will be treated according to standard care. The “prematurity ROP group” will be defined as patients with infants developing ROP type 1 or 2 (visible on fundus examination after 4 weeks of life and at least 31 GA) and born before 29 GA. The “prematurity no-ROP group” will be defined as patients with infants without any stage of ROP and born before 29 GA. The “full-term no ROP group” will comprise patients with infants without ROP and born at full-term. Mother-infant dyads in life-threatening conditions will not be included (Table 1).

**Table 1:** Inclusion and exclusion criteria

Inclusion criteria	Exclusion criteria
Mothers delivering a premature newborn of less than 29 weeks’ gestational age (GA) after obtaining their non-opposition	Mothers giving birth between 29 and 38 GA + 6 days
Mothers giving birth at full term between 39 and 41 GA + 6 days, after obtaining their non-opposition	Mothers with a vital prognosis
The mother must be of legal age	The mother is not affiliated with a social security system
The mother must not be under legal guardianship	For full-term mothers: Current or previous history of an obstetrical pathology of any origin (vascular such as gestational hypertension, pre-eclampsia; gestational diabetes; intrauterine growth retardation, maternal infection during pregnancy such as toxoplasmosis, cytomegalovirus, rubella, measles, chickenpox)

### Sample size calculation

The sample size in this study was calculated using multiple regression to detect a difference of 3 in the mean plasma FA receptor level between groups with 80% power (α error = 0.05), a ratio of 2:1 between groups, and a coefficient of determination R^2^ of 0.10. Overall, a sample size of 100 mother-infant dyads is required: 70 mothers over 18 years of age giving birth before 29 GA and 30 mothers with full-term pregnancies (39–41 GA + 6 days).

### Assessment of variables

#### Maternal history collection

Maternal history that may cause changes in placental function will be recorded, for example, pregnancy-induced hypertension, pre-eclampsia, abruptio placentae, and autoimmune and thromboembolic diseases during the pregnancy (Table 2). The investigator will give this document to the patient following delivery.

**Table 2:** Maternal characteristics

**Inclusion number**	
**Age**	
**Pre-pregnancy weight (kg)**	
**End of pregnancy weight (kg)**	
**Size (cm)**	
**Smoking (during pregnancy)**	
**Alcohol (during pregnancy)**	
**Taking medication**	
**Multiple pregnancies?**	
**Delivery route**	
**Primipara?**	
**Diabetes (before pregnancy), if yes, HbA1c (%)**	
**Dyslipidemia**	
**Arterial hypertension**	
**Digestive diseases (please specify)**	
**Thromboembolic diseases**	
**Dysimmune diseases**	
**Obstetrical history (please specify)**	
Events during this pregnancy	
**Recurrent vomiting (what trimester? how often?)**	
**Gestational diabetes (specify if insulin is required)**	
**Maternal–fetal infection (specify)**	
**Gestational hypertension**	
**Pre-eclampsia**	
**Eclampsia**	
**Premature delivery risks**	
**Placental characteristics**	Birth weight (g): Weight at pathology analysis (g): Hypotrophic? Immature? Hematoma? Placental ischemia? Infection? (chorioamnionitis or funiculitis?)

#### Newborn follow-up

ROP screening will be performed with a wide-field RETCAM II camera (Clarity Medical Systems; Pleasanton, CA, USA) using a lid speculum after application of a local anesthetic (oxybuprocaine hydrochloride 1.6 mg/0.4 mL; Thea Laboratories, Clermont-Ferrand, France) only in premature infants because ROP does not occur in full-term infants. Pupillary dilation will be performed before hand using one drop of 2.5% epinephrine (phenylephrine 5% diluted to 2.5%; Europhta Laboratories, Monaco) and one drop of tropicamide (2 mg/0.4 mL; Thea Laboratories, Clermont-Ferrand, France). A trained nurse will complete the procedure, and a trained pediatrics-specialized ophthalmologist will analyze all fundus photographs. The initiation of ROP screening should be based on the infant’s postmenstrual age because the onset of severe ROP correlates better with postmenstrual age than with postnatal age [2]. The screening will begin at 4th weeks of life but never before 31 weeks of postconceptional age (PCA) and will be repeated every other week until 41 weeks’ PCA if no ROP was detected, and every week and up to twice a week in the case of ROP. ROP staging will be determined according to the International Classification of ROP consensus statement [37] (Table 3).

**Table 3:** The International Classification of Retinopathy of Prematurity [37]

**Stage**		**Localization**		**Severity**	
1	Demarcation line	I	Circle area centered on the optic nerve with a radius twice the distance from the optic nerve to the macula	Plus disease	Sufficient vascular dilatation and tortuosity in at least 2 quadrants of the eye
2	Ridge	II	It extends from the end of zone I to the nasal ora serrata	Pre-plus disease	Insufficient vascular dilatation and tortuosity less than in plus disease
3	Extraretinal fibrovascular proliferation	III	It corresponds to the growing remaining crescent area		
4a	Extrafoveal partial retinal detachment				
4b	Foveal partial retinal detachment				
5	Total retinal detachment				

If necessary, transpupillary laser treatment of the ischemic areas will be performed under general anesthesia. This treatment will be carried out at the Fondation Ophtalmologique Adolphe de Rothschild, Paris, France; the treatment involves " + " attacks in zone I, stage 3 in zone I, and stages 2 and 3 " + " in zone II [38]. Significant risk factors for developing ROP, namely, term and weight at birth, duration of mechanical ventilation, sepsis, use of erythropoietin, red blood cell transfusion, and cerebral hemorrhage will be documented during the first month of life (Table 4).

**Table 4:** Newborn characteristics

**Inclusion number**	
Date of birth	
Term of birth (GA)	
Birth weight (g)	
Fetal growth restriction	< 10th percentile? < 5th percentile? Causes: Maternal? Fetal? Placental?
Weight to W1/W2/W3/W4 (g)	
Gender	Male / Female
Oxygen therapy:	Total duration (days): Duration of mechanical ventilation (days): Duration of CPAP (days): Duration of HFNC (days): Duration of simple nasal cannula (days): Maximum FiO_2_ (%):
Sepsis	Yes/No
Necrotizing enterocolitis	Stage I / II / III? Required surgical management? Yes/No
Neurologic conditions	Intracranial hemorrhage (specify the grade): Periventricular hemorrhage (specify the grade): Subependymal hemorrhage (specify the grade): Periventricular leukomalacia (specify the grade):
Anemia	Minimum hemoglobin level: Transfusion (specify the number of units and quantity): EPO use?
Ophthalmologic follow-up	Date of first fundus: Week of life at first fundus: Week of gestation at first fundus: Date of fundus at first sign of ROP: Week of life at first sign of ROP: Week of gestation at first sign of ROP: Stage of ROP at first sign of ROP: Localization of ROP at first sign of ROP: Stage plus or pre-plus: ROP type 1 or 2: Most severe eye: Date of fundus at maximal ROP: Week of life at maximal ROP: Week of gestation at maximal ROP: Number of fundus before first sign of ROP: Number of fundus before maximal ROP: Total number of fundus with RETCAM: Laser treatment (which eye?): Date of laser treatment: Intravitreal treatment (bevacizumab) (which eye?): Date of intravitreal treatment:
Date of discharge (specify if transferred to another hospital)	

### Biochemical assessments

#### Maternal samples

A 0.5-mL cord blood sample will be collected by venipuncture at the time of delivery in an EDTA tube by a midwife or a gynecologist. A 5-mL blood sample will also be taken from mothers within a maximum delay of 2 days following delivery. Red blood cells will be immediately separated from serum, and samples will be stored at − 80 °C until lipidomic analyses. Three placenta samples (1 cm wide by 1 cm long) will be cut from mothers after delivery and stored at − 80 °C. Following the recommendations of the Ethical considerations for clinical trials on medicinal products conducted with minors,” the volume of blood collected will be limited to 0.5 mL for the umbilical cord blood and 5 mL for the mother’s venous blood [39].

#### Lipidomic blood analyses

The red blood cell FA composition for maternal and cord blood samples will be determined according to previously described procedures [36, 40]: Total lipids are extracted from erythrocytes, according to Moilanen and Nikkari [41]. Phospholipids are purified from total lipid extracts using silica cartridges [42], and transmethylated using boron trifluoride in methanol [43]. The fatty acid methyl esters (FAMEs) and dimethyl acetals (DMAs) are extracted with hexane and analyzed on a Hewlett Packard Model 5890 gas chromatograph using a CPSIL-88 column (100 mm × 0.25 mm i.d., film thickness 0.20 mm; Varian, Les Ulis, France) equipped with a flame ionization detector. Hydrogen is used as the carrier gas (inlet pressure 210 kPa). The oven temperature is held at 60 °C for 5 min, increased to 165 °C at 15 °C/min, held for 1 min, and then increased to 225 °C at 2 °C/min and finally kept at 225 °C for 17 min. The injector and the detector are maintained at 250 °C. FAMEs are identified by comparison with commercial and synthetic standards. The data will be processed using the EZChrom Elite software (Agilent Technologies, Massy, France) and reported as a percentage of the total FAMEs and DMAs [36, 40].

#### Placental lipid transport/receptor protein analyses

Proteins will be identified by Western blotting (WB). Tissues are previously treated with a modified RIPA buffer and stored at -80 °C. The samples are denatured by adding Laemmli (Bio-Rad, Hercules, CA, USA) 10 × low glycerol concentration. Proteins are deposited in each well (20 μg for each of the experimental conditions) and then separated according to their molecular weight by migration on an SDS Stain-Free acrylamide gel (Mini-PROTEAN TGX, Stain-Free 4–15% Gels, Bio-Rad, Hercules, CA, USA) at 70 V for 15 min, and then at 140 V for approximately 1 h. Proteins are then transferred to a 0.2-μm nitrocellulose membrane (reference 1,620,112, Bio-Rad, Hercules, CA, USA) using the Trans-Blot Turbo Transfer System (Bio-Rad, Hercules, CA, USA) in semi-dry condition at 2.5 A and 25 V for 7 min. The non-specific sites are saturated by incubating the membrane for 1 h at room temperature in 1% PBS-Tween 5% skimmed milk The proteins of interest are then revealed by the successive incubation of a primary antibody and then a secondary antibody. The primary antibodies specific to the protein of interest are diluted 1/1000 in 1% PBS-Tween 5% skimmed milk buffer and incubated overnight at + 4 °C. The secondary antibody specific to the primary antibody (polyclonal goat anti-mouse / HRP, Dako, reference P0447), coupled to a peroxidase (HRP) is diluted 1/1000 in 1% PBS-Tween 5% skimmed milk and then incubated for 1 h at room temperature. Chemiluminescent protein detection (ECL) is performed using a ChemiDocTM XRS reader with the Western lightning® Plus-ECL Enhanced Chemiluminescence Substrate (NEL 104001EA, Perkin-Elmer). Quantification of proteins of interest will be performed using the ImageLab® v.4.0.1 software.

## Objectives and statistical analysis

### Outcomes

The primary outcome is to evaluate the relationship between the expression level of placental FA receptors and ROP occurrence.

Secondary outcomes of the study are: (1) assessment of the relationship between blood cord FA content and the expression level of placental FA receptors; (2) assessment of the relationship between maternal blood FA content and the expression level of placental FA receptors; (3) assessment of the relationship between placental FA receptor expression levels and the occurrence and severity of ROP.

## Statistical methods

The data will be reviewed at the end of the study before statistical analysis. Statistical analysis will be performed using STATA v15.1 (StataCorp, College Station, TX, USA).

Quantitative data will be expressed as median and interquartile range [IQR]. The groups will be compared for quantitative variables using parametric or non-parametric tests according to normal/abnormal data distribution, and for qualitative variables using the chi-square test or Fisher’s exact test. Spearman correlations will be used to analyze the extent to which the placental FA receptor expression is associated with ROP occurrence. The extent to which the placental FA receptor expression is associated with the maternal blood FA level and the blood cord FA level will be analyzed through Spearman correlations. Linear regression analyses will be carried out to compare placental FA receptor expression levels as a function of GA. Statistical significance is to be set at *p* < 0.05, and the tests will be two-tailed.

## Ethical, regulatory, and dissemination aspects

The clinical study will be conducted in accordance with the relevant versions of the French and European laws (no. 2021–300 of 5 March 2021 related to research involving humans, amended by order no. 2016–800 of 16 June 2016 and its implementing decrees), the Declaration of Helsinki, and the recommendations of Good Clinical Practice.

In accordance with article L1121-1 of the Public Health Code, this study constitutes research of category 3 involving human participants, in that it constitutes risk-free research in which all procedures are performed and products are used in the standard way.

This clinical study was submitted to and approved by the Ethical Review Board of Dijon (*Comité de Protection des Personnes* – CPP Ouest III—Dijon) on 7 February 2021, and two amendments were submitted and approved on 30 August 2021 (new inclusion site added) and 24 August 2022 (extension of inclusion time).

Requests for substantial modifications should be addressed by the sponsor for approval or notification to the Ethical Review Board concerned in compliance with law 2021–300 of 5 March 2012 and its implementing decrees.

All the submissions/declarations were made by the Sponsor Department at CHU Dijon, which manages the quality of the data collected. The data collected during the study will be processed electronically in accordance with the requirements of the CNIL, the French Data Protection Authority (in compliance with the French Reference Methodology MR003).

As required, the sponsor has provided an insurance policy to cover the financial consequences of its civil liability following the regulations.

It has been possible to carry out the protocol and the trial thanks to C. Renaud, project manager of the clinical research and innovation office; Prof. Creuzot Garcher, MD, PhD Head, Department of Ophthalmology; Prof. Simon, MD, PhD, Department of Gynecology, Dijon University Hospital, France; and Dr. Acar, group leader of the Eye and Nutrition Research Group, INRAE, Burgundy University. Also, the midwives study coordinator of the Gynecology Department at Dijon University Hospital and Dr. Carré, Department of Ophthalmology, coordinated the inclusion of the patients and collection of the study data. The study was converted to a multicenter research thanks to the collaboration of the departments of Ophthalmology (Dr. A. Daruich), Gynecology (Dr. M. Driessen), and Neonatology (Dr. E. Kermorvant) at Necker Hospital, Paris.

The trial results will be published in international ophthalmological, medical, and scientific journals. The investigators, who will share the entirety of the final trial dataset, will follow the rules and guidelines of the International Committee for Medical Journal Editors (ICMJE).

Per the provisions of Article R5121-13 of the Public Health Code, the investigator and any person called upon to collaborate in the studies are bound by professional secrecy, particularly concerning the nature of the products studied, the studies, the persons involved, and the results obtained subject to the provisions of Article L1123-14 of the Public Health Code.

Without the promoter’s agreement (Dijon University Hospital), they may not provide information on the study to anyone other than the Health Authorities, including the inspectors, as mentioned in article R5121-13 of the Public Health Code.

The study will not be commented on, either orally or in writing, without joint authorization from the coordinating investigator and the promoter (Dijon University Hospital). Medical data concerning patients will only be communicated to the promoter and, if necessary, to the authorized health authorities under conditions that guarantee patient confidentiality. Patients may exercise their rights of access and rectification with their investigator.

At the end of the study, all documents related to the study (including copies of the case report forms) should be archived at the study site or a centralized archiving site. Particular attention should be paid to identifying the patients included in the trial and the consent forms. This list and the forms are the most critical documents to be archived by the investigator.

All documents related to the study should be kept for 15 years after the end of the study. At the end of this period, the sponsor will be informed by the investigators that archiving has been completed.

## Discussion

PUFAs from the omega-3 series are strong regulators of perinatal retinal vascular development. It is now well established that n-3 LC-PUFAs play crucial roles in retinal angiogenesis and vascularization [44], limit the severity of pathological retinal neovascularization, and improve the regression of ocular vascular lesions [45]. To our knowledge, there are limited data available on lipid or LC-PUFA status in preterm infants developing ROP. Nutrient transport across the placenta and into fetal circulation is complex. Like glucose or amino acids, FAs are an essential macronutrient for adequate fetal growth and they cross the syncytiotrophoblast through specific transporters [46]. FATPs and FAT CD36 are integral membrane proteins that are important for the uptake of LC-PUFA [47]. FATP1 and FATP4 are mostly studied in placental tissue as their expression is correlated with DHA levels in maternal plasma, cord blood, and placental phospholipids, suggesting an important role in the transfer of LC-PUFA [33]. All of the n-6 and n-3 FAs accumulated by the fetus must be derived from the mother via LC-PUFA placental transfer [48]. Therefore, the diet must provide this after birth [28]. Previously, Pallot and colleagues in the Omega-ROP study were the first to characterize erythrocyte lipids in preterm infants born before 29 weeks of gestational age (GA) and showed abnormalities in placental PUFA transfer in preterm infants who will develop ROP [32]. DHA levels in erythrocytes were shown to range between 5.5 and 5.8% of total FAs in mothers who delivered full-term infants (39–41 GA) [49] and were reported to be reduced to 2.5% of total FAs in mothers of 33 GA preterm infants [50].

We hypothesize that in utero bioavailability of n-6 and n-3 LC-PUFAs could influence the occurrence of ROP. Mechanistic studies are needed to determine whether dysfunction in the placental transfer of n-3 LC-PUFAs is involved in the maternal and fetal erythrocyte FA modifications observed. In the OmegaROP-2 study, we aim to evaluate the association between the expression level of placental FA receptors and the development of ROP.

## Trial status

This trial is ongoing, and patient inclusion is not yet complete. The first patient was included on 20 April 2021. Initially, recruitment by the investigating center was planned until 20 October 2022, and the study period was to end in February 2023. However, due to the low incidence of births before 29 GA, this protocol has been amended: We have received CPP approval to extend the inclusion period by 24 months and make it a multicenter study with the collaboration of the ophthalmology and gynecology departments of Necker Hospital, Paris, France.

## Data Availability

Not applicable. Upon completion of the study, de-identified data will be made by request available per the funder.
